# Ethyl 2-(2-methyl-1*H*-benzimidazol-1-yl)acetate

**DOI:** 10.1107/S160053680802672X

**Published:** 2008-08-23

**Authors:** Guang-Hai Xu, Wei Wang

**Affiliations:** a Ordered Matter Science Research Center, Southeast University, Nanjing 210096, People’s Republic of China

## Abstract

A new benzimidazole compound, C_12_H_14_N_2_O_2_, has been synthesized by the reaction of 2-methyl-1*H*-benzimidazole and ethyl 2-bromo­acetate. In the crystal structure, weak inter­molecular C—H⋯N hydrogen bonds link the mol­ecules into chains. π⋯π Contacts (centroid⋯centroid distance = 3.713 Å) are observed. A C—H⋯π inter­action is also present. The N—C—C—O torsion angle is 178.4 (2)°.

## Related literature

For related literature, see: Aaker *et al.* (2005 ▶).
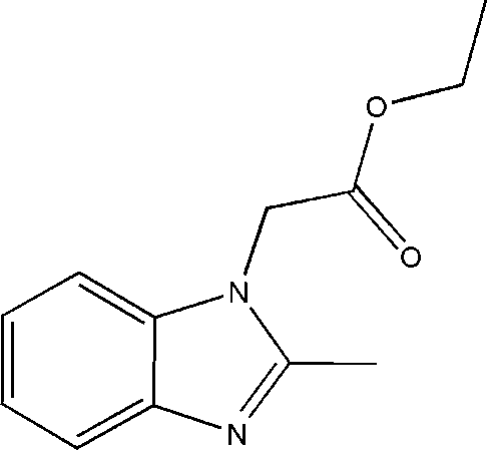


## Experimental

### 

#### Crystal data

C_12_H_14_N_2_O_2_
*M* *_r_* = 218.25
Monoclinic, 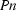

*a* = 10.854 (2) Å
*b* = 4.7959 (10) Å
*c* = 11.842 (2) Å
β = 111.42 (3)°
*V* = 573.9 (2) Å^3^
*Z* = 2
Mo *K*α radiation
μ = 0.09 mm^−1^
*T* = 295 (2) K
0.2 × 0.1 × 0.1 mm

#### Data collection

Rigaku SCXmini diffractometer
Absorption correction: multi-scan (*CrystalClear*; Rigaku, 2005 ▶) *T*_min_ = 0.990, *T*_max_ = 1.000 (expected range = 0.981–0.991)
5696 measured reflections
1323 independent reflections
1085 reflections with *I* > 2σ(*I*)
*R*_int_ = 0.033

#### Refinement

*R*[*F* ^2^ > 2σ(*F* ^2^)] = 0.041
*wR*(*F* ^2^) = 0.089
*S* = 1.12
1323 reflections
145 parameters
2 restraints
H-atom parameters constrained
Δρ_max_ = 0.10 e Å^−3^
Δρ_min_ = −0.15 e Å^−3^

### 

Data collection: *CrystalClear* (Rigaku, 2005 ▶); cell refinement: *CrystalClear*; data reduction: *CrystalClear*; program(s) used to solve structure: *SHELXS97* (Sheldrick, 2008 ▶); program(s) used to refine structure: *SHELXL97* (Sheldrick, 2008 ▶); molecular graphics: *SHELXTL* (Sheldrick, 2008 ▶); software used to prepare material for publication: *SHELXTL*.

## Supplementary Material

Crystal structure: contains datablocks I, New_Global_Publ_Block. DOI: 10.1107/S160053680802672X/wn2276sup1.cif

Structure factors: contains datablocks I. DOI: 10.1107/S160053680802672X/wn2276Isup2.hkl

Additional supplementary materials:  crystallographic information; 3D view; checkCIF report

## Figures and Tables

**Table 1:** Hydrogen-bond geometry (Å, °)

*D*—H⋯*A*	*D*—H	H⋯*A*	*D*⋯*A*	*D*—H⋯*A*
C9—H9*B*⋯N1^i^	0.97	2.61	3.532 (3)	159
C8—H8*C*⋯*Cg*1^ii^	0.97	2.74	3.633 (5)	155

Symmetry codes: (i) 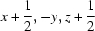; (ii) 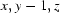. *Cg*1 is the centroid of the imidazole ring.

**Fig. 1.**
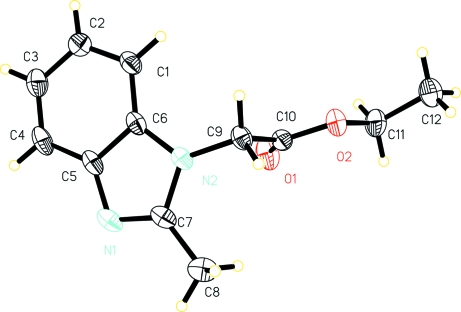
The molecular structure of the title compound, with the atomic numbering scheme. Displacement ellipsoids are drawn at the 30% probability level.

**Fig. 2.**
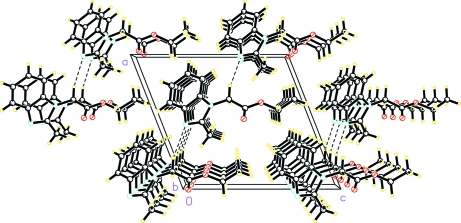
A view of the packing of the title compound, with pi···pi stacking along the b axis. Dashed lines indicate hydrogen bonds.

## supplementary crystallographic information

### Comment

The molecular structure of the title compound is shown in Fig. 1. The benzimidazole system is essentially planar, with a dihedral angle of 0.88 (14)° between the planes of the benzene and imidazole rings. The N2—C9—C10—O2 torsion angle is 178.4 (2)°.

In the crystal structure, molecules are connected by weak intermolecular C—H···N hydrogen bonds, forming a polymeric chain (see Table 1 and Fig. 2). A C—H···π contact (see Table 1, Cg1 is the centroid of the imidazole ring) and π···π stacking (centroid···centroid distance = 3.713 Å) between neighboring benzimidazoles further stabilize the structure.

### Experimental

The synthesis of 2-methyl-1*H*-benzimidazole was reported previously (Aaker *et al.*, 2005). Ethyl 2-bromoacetate (1.65 g, 10 mmol) was added to a solution of 2-methyl-1*H*-benzimidazole (1.32 g, 10 mmol) and NaH (0.6 g, 26 mmol) in THF (30 ml). After the mixture was stirred for 12 h at room temperature, the precipitate was filtered off and the solution was evaporated in vacuum. The crude product was then crystallized from ethanol. Single crystals suitable for X-ray analysis were obtained by slow evaporation of an ethanol solution.

### Refinement

All H atoms were positioned geometrically and were allowed to ride on the atoms to which they are bonded. C—H = 0.93–0.97 Å; *U*_iso_(H) = x*U*_eq_(C), where x = 1.5 for methyl and x = 1.2 for all other H atoms.

### Crystal data

**Table:**

C_12_H_14_N_2_O_2_	*F*_000_ = 232
*M**_r_* = 218.25	*D*_x_ = 1.263 Mg m^−^^3^
Monoclinic, *P**n*	Mo *K*α radiation λ = 0.71073 Å
Hall symbol: P -2yac	Cell parameters from 6060 reflections
*a* = 10.854 (2) Å	θ = 6.4–55.1º
*b* = 4.7959 (10) Å	µ = 0.09 mm^−^^1^
*c* = 11.842 (2) Å	*T* = 295 (2) K
β = 111.42 (3)º	Prism, colorless
*V* = 573.9 (2) Å^3^	0.2 × 0.1 × 0.1 mm
*Z* = 2	

### Data collection

**Table:**

Rigaku SCXmini diffractometer	1323 independent reflections
Radiation source: fine-focus sealed tube	1085 reflections with *I* > 2σ(*I*)
Monochromator: graphite	*R*_int_ = 0.033
Detector resolution: 13.6612 pixels mm^-1^	θ_max_ = 27.5º
*T* = 294(2) K	θ_min_ = 3.2º
CCD_Profile_fitting scans	*h* = −14→14
Absorption correction: multi-scan(CrystalClear; Rigaku, 2005)	*k* = −6→6
*T*_min_ = 0.990, *T*_max_ = 1.000	*l* = −15→15
5696 measured reflections	

### Refinement

**Table:**

Refinement on *F*^2^	Secondary atom site location: difference Fourier map
Least-squares matrix: full	Hydrogen site location: inferred from neighbouring sites
*R*[*F*^2^ > 2σ(*F*^2^)] = 0.041	H-atom parameters constrained
*wR*(*F*^2^) = 0.090	*w* = 1/[σ^2^(*F*_o_^2^) + (0.0435*P*)^2^ + 0.018*P*] where *P* = (*F*_o_^2^ + 2*F*_c_^2^)/3
*S* = 1.12	(Δ/σ)_max_ < 0.001
1323 reflections	Δρ_max_ = 0.10 e Å^−^^3^
145 parameters	Δρ_min_ = −0.15 e Å^−^^3^
2 restraints	Extinction correction: none
Primary atom site location: structure-invariant direct methods	

### Special details

**Table:**

Geometry. All e.s.d.'s (except the e.s.d. in the dihedral angle between two l.s. planes) are estimated using the full covariance matrix. The cell e.s.d.'s are taken into account individually in the estimation of e.s.d.'s in distances, angles and torsion angles; correlations between e.s.d.'s in cell parameters are only used when they are defined by crystal symmetry. An approximate (isotropic) treatment of cell e.s.d.'s is used for estimating e.s.d.'s involving l.s. planes.
Refinement. Refinement of *F*^2^ against ALL reflections. The weighted *R*-factor *wR* and goodness of fit *S* are based on *F*^2^, conventional *R*-factors *R* are based on *F*, with *F* set to zero for negative *F*^2^. The threshold expression of *F*^2^ > 2σ(*F*^2^) is used only for calculating *R*-factors(gt) *etc*. and is not relevant to the choice of reflections for refinement. *R*-factors based on *F*^2^ are statistically about twice as large as those based on *F*, and *R*- factors based on ALL data will be even larger.

### Fractional atomic coordinates and isotropic or equivalent isotropic displacement parameters (Å^2^)

**Table:**

	*x*	*y*	*z*	*U*_iso_*/*U*_eq_	
O2	0.14135 (17)	0.1457 (4)	0.20123 (14)	0.0515 (5)	
C5	0.1006 (3)	−0.1654 (5)	−0.2701 (2)	0.0449 (6)	
C1	0.3029 (2)	−0.2567 (6)	−0.0948 (2)	0.0471 (6)	
H1A	0.3572	−0.2219	−0.0148	0.056*	
N2	0.12080 (19)	0.0836 (4)	−0.10788 (17)	0.0412 (5)	
C10	0.1019 (2)	0.0363 (5)	0.0906 (2)	0.0413 (5)	
C9	0.1639 (2)	0.1907 (6)	0.0146 (2)	0.0425 (6)	
H9A	0.1409	0.3867	0.0121	0.051*	
H9B	0.2594	0.1753	0.0518	0.051*	
N1	−0.0107 (2)	0.0004 (5)	−0.30007 (18)	0.0497 (6)	
O1	0.0293 (2)	−0.1587 (4)	0.05777 (18)	0.0685 (6)	
C4	0.1383 (3)	−0.3615 (6)	−0.3381 (2)	0.0576 (8)	
H4A	0.0850	−0.3960	−0.4184	0.069*	
C6	0.1845 (2)	−0.1163 (5)	−0.1499 (2)	0.0390 (6)	
C7	0.0038 (2)	0.1447 (5)	−0.2017 (2)	0.0454 (6)	
C2	0.3361 (3)	−0.4510 (7)	−0.1649 (2)	0.0540 (7)	
H2A	0.4147	−0.5501	−0.1311	0.065*	
C3	0.2550 (3)	−0.5027 (7)	−0.2850 (3)	0.0596 (7)	
H3A	0.2805	−0.6350	−0.3296	0.072*	
C12	0.1640 (3)	0.1400 (8)	0.4080 (3)	0.0737 (10)	
H12A	0.1322	0.0602	0.4666	0.111*	
H12B	0.1510	0.3383	0.4051	0.111*	
H12C	0.2565	0.0999	0.4306	0.111*	
C8	−0.0919 (3)	0.3503 (6)	−0.1883 (3)	0.0598 (8)	
H8A	−0.1666	0.3635	−0.2632	0.090*	
H8B	−0.0500	0.5293	−0.1683	0.090*	
H8C	−0.1212	0.2914	−0.1248	0.090*	
C11	0.0894 (3)	0.0179 (7)	0.2857 (3)	0.0617 (8)	
H11A	0.1016	−0.1826	0.2873	0.074*	
H11B	−0.0044	0.0568	0.2617	0.074*	

### Atomic displacement parameters (Å^2^)

**Table:**

	*U*^11^	*U*^22^	*U*^33^	*U*^12^	*U*^13^	*U*^23^
O2	0.0623 (12)	0.0550 (11)	0.0392 (10)	−0.0084 (9)	0.0209 (9)	−0.0061 (8)
C5	0.0512 (15)	0.0505 (15)	0.0271 (11)	−0.0146 (12)	0.0075 (11)	0.0011 (11)
C1	0.0448 (14)	0.0577 (16)	0.0327 (12)	−0.0087 (12)	0.0070 (10)	−0.0014 (12)
N2	0.0423 (11)	0.0447 (12)	0.0312 (10)	−0.0065 (9)	0.0069 (8)	−0.0003 (9)
C10	0.0419 (13)	0.0418 (13)	0.0383 (13)	0.0019 (11)	0.0126 (10)	−0.0012 (11)
C9	0.0452 (14)	0.0437 (15)	0.0345 (12)	−0.0095 (11)	0.0095 (10)	−0.0074 (10)
N1	0.0493 (12)	0.0502 (13)	0.0363 (12)	−0.0092 (11)	0.0000 (9)	0.0043 (10)
O1	0.0865 (15)	0.0659 (14)	0.0564 (12)	−0.0334 (12)	0.0301 (11)	−0.0148 (10)
C4	0.0744 (19)	0.0620 (18)	0.0291 (13)	−0.0186 (16)	0.0102 (13)	−0.0110 (13)
C6	0.0432 (13)	0.0434 (14)	0.0283 (12)	−0.0123 (11)	0.0104 (10)	0.0010 (10)
C7	0.0426 (14)	0.0450 (14)	0.0395 (14)	−0.0087 (12)	0.0041 (11)	0.0077 (11)
C2	0.0499 (15)	0.0627 (19)	0.0483 (17)	0.0003 (14)	0.0166 (13)	−0.0013 (14)
C3	0.0726 (19)	0.0613 (18)	0.0468 (17)	−0.0082 (15)	0.0242 (15)	−0.0129 (14)
C12	0.083 (2)	0.095 (3)	0.0472 (17)	0.0068 (19)	0.0283 (16)	0.0017 (17)
C8	0.0515 (16)	0.0546 (16)	0.0659 (19)	0.0001 (14)	0.0127 (14)	0.0089 (14)
C11	0.0716 (19)	0.073 (2)	0.0508 (18)	−0.0011 (16)	0.0346 (15)	−0.0009 (15)

### Geometric parameters (Å, °)

**Table:**

O2—C10	1.329 (3)	C4—C3	1.370 (5)
O2—C11	1.451 (3)	C4—H4A	0.9300
C5—N1	1.380 (4)	C7—C8	1.482 (4)
C5—C4	1.393 (4)	C2—C3	1.395 (4)
C5—C6	1.401 (3)	C2—H2A	0.9300
C1—C2	1.380 (4)	C3—H3A	0.9300
C1—C6	1.385 (4)	C12—C11	1.497 (4)
C1—H1A	0.9300	C12—H12A	0.9600
N2—C6	1.376 (3)	C12—H12B	0.9600
N2—C7	1.379 (3)	C12—H12C	0.9600
N2—C9	1.446 (3)	C8—H8A	0.9600
C10—O1	1.193 (3)	C8—H8B	0.9600
C10—C9	1.502 (3)	C8—H8C	0.9600
C9—H9A	0.9700	C11—H11A	0.9700
C9—H9B	0.9700	C11—H11B	0.9700
N1—C7	1.315 (3)		
			
C10—O2—C11	116.5 (2)	N1—C7—C8	125.7 (2)
N1—C5—C4	130.8 (2)	N2—C7—C8	122.1 (2)
N1—C5—C6	110.3 (2)	C1—C2—C3	121.8 (3)
C4—C5—C6	118.8 (3)	C1—C2—H2A	119.1
C2—C1—C6	116.5 (2)	C3—C2—H2A	119.1
C2—C1—H1A	121.7	C4—C3—C2	120.9 (3)
C6—C1—H1A	121.7	C4—C3—H3A	119.5
C6—N2—C7	107.06 (19)	C2—C3—H3A	119.5
C6—N2—C9	126.07 (19)	C11—C12—H12A	109.5
C7—N2—C9	126.7 (2)	C11—C12—H12B	109.5
O1—C10—O2	124.6 (2)	H12A—C12—H12B	109.5
O1—C10—C9	125.3 (2)	C11—C12—H12C	109.5
O2—C10—C9	110.04 (19)	H12A—C12—H12C	109.5
N2—C9—C10	112.00 (19)	H12B—C12—H12C	109.5
N2—C9—H9A	109.2	C7—C8—H8A	109.5
C10—C9—H9A	109.2	C7—C8—H8B	109.5
N2—C9—H9B	109.2	H8A—C8—H8B	109.5
C10—C9—H9B	109.2	C7—C8—H8C	109.5
H9A—C9—H9B	107.9	H8A—C8—H8C	109.5
C7—N1—C5	105.4 (2)	H8B—C8—H8C	109.5
C3—C4—C5	119.0 (3)	O2—C11—C12	107.0 (3)
C3—C4—H4A	120.5	O2—C11—H11A	110.3
C5—C4—H4A	120.5	C12—C11—H11A	110.3
N2—C6—C1	132.1 (2)	O2—C11—H11B	110.3
N2—C6—C5	105.0 (2)	C12—C11—H11B	110.3
C1—C6—C5	122.9 (2)	H11A—C11—H11B	108.6
N1—C7—N2	112.2 (2)		
			
C11—O2—C10—O1	−1.1 (4)	C2—C1—C6—C5	0.2 (3)
C11—O2—C10—C9	180.0 (2)	N1—C5—C6—N2	0.1 (3)
C6—N2—C9—C10	−93.6 (3)	C4—C5—C6—N2	−179.8 (2)
C7—N2—C9—C10	80.8 (3)	N1—C5—C6—C1	179.1 (2)
O1—C10—C9—N2	2.6 (4)	C4—C5—C6—C1	−0.8 (4)
O2—C10—C9—N2	−178.4 (2)	C5—N1—C7—N2	0.6 (3)
C4—C5—N1—C7	179.5 (3)	C5—N1—C7—C8	−179.1 (2)
C6—C5—N1—C7	−0.4 (3)	C6—N2—C7—N1	−0.6 (3)
N1—C5—C4—C3	−179.0 (3)	C9—N2—C7—N1	−175.9 (2)
C6—C5—C4—C3	0.9 (4)	C6—N2—C7—C8	179.1 (2)
C7—N2—C6—C1	−178.6 (3)	C9—N2—C7—C8	3.9 (4)
C9—N2—C6—C1	−3.3 (4)	C6—C1—C2—C3	0.2 (4)
C7—N2—C6—C5	0.3 (2)	C5—C4—C3—C2	−0.5 (4)
C9—N2—C6—C5	175.6 (2)	C1—C2—C3—C4	−0.1 (4)
C2—C1—C6—N2	179.0 (3)	C10—O2—C11—C12	170.5 (2)

### Hydrogen-bond geometry (Å, °)

**Table:**

*D*—H···*A*	*D*—H	H···*A*	*D*···*A*	*D*—H···*A*
C9—H9B···N1^i^	0.97	2.61	3.532 (3)	159
C8—H8C···Cg1^ii^	0.97	2.74	3.633 (5)	155

Symmetry codes: (i) *x*+1/2, −*y*, *z*+1/2; (ii) *x*, *y*−1, *z*.
